# The Impact of Particulate Matter (PM2.5) on Human Retinal Development in hESC-Derived Retinal Organoids

**DOI:** 10.3389/fcell.2021.607341

**Published:** 2021-02-12

**Authors:** Yuxiao Zeng, Minghui Li, Ting Zou, Xi Chen, Qiyou Li, Yijian Li, Lingling Ge, Siyu Chen, Haiwei Xu

**Affiliations:** ^1^Southwest Hospital/Southwest Eye Hospital, Third Military Medical University (Army Medical University), Chongqing, China; ^2^Key Lab of Visual Damage and Regeneration & Restoration of Chongqing, Chongqing, China; ^3^Department of Ophthalmology, Beijing Friendship Hospital, Capital Medical University, Beijing, China

**Keywords:** PM2.5, hESC-derived retinal organoids, neural retina, proliferation, apoptosis

## Abstract

Increasing evidence demonstrated that PM2.5 could cross the placenta and fetal blood–brain barrier, causing neurotoxicity of embryonic development. The retina, an embryologic extension of the central nervous system, is extremely sensitive and vulnerable to environmental insults. The adverse effects of PM2.5 exposure on the retina during embryonic neurodevelopment are still largely unknown. Our goal was to investigate the effect of PM2.5 on human retinal development, which was recapitulated by human embryonic stem cell (hESC)-derived retinal organoids (hEROs). In the present study, using the hEROs as the model, the influences and the mechanisms of PM2.5 on the developing retina were analyzed. It demonstrated that the formation rate of the hERO-derived neural retina (NR) was affected by PM2.5 in a concentration dosage-dependent manner. The areas of hEROs and the thickness of hERO-NRs were significantly reduced after PM2.5 exposure at the concentration of 25, 50, and 100 μg/ml, which was due to the decrease of proliferation and the increase of apoptosis. Although we did not spot significant effects on retinal differentiation, PM2.5 exposure did lead to hERO-NR cell disarranging and structural disorder, especially retinal ganglion cell dislocation. Transcriptome analysis showed that PM2.5 treatment was significantly associated with the mitogen-activated protein kinase (MAPK) and phosphoinositide 3-kinase (PI3K)/AKT pathways and reduced the level of the fibroblast growth factors (FGFs), particularly FGF8 and FGF10. These results provided evidence that PM2.5 exposure potentially inhibited proliferation and increased apoptosis at the early development stage of the human NR, probably through the MAPK and PI3K/Akt pathway. Our study suggested that exposure to PM2.5 suppressed cell proliferation and promoted cell apoptosis, thereby contributing to abnormal human retinal development.

## Introduction

Air pollution has caused extensive acute and chronic health issues (Cohen et al., 2017; Miyazaki et al., 2019). The polluted air represents a mixture of various substances, including particulate matter (PM), gases, and biological molecules (Bulot et al., 2019; Song et al., 2019), which affect human health, in addition to direct inhalation. Among them, fine particles (particles ≤ 2.5 mm; PM2.5) are regarded as a great health threat and are closely related to diseases, such as cardiovascular and respiratory diseases (Cohen et al., 2017; Sugiyama et al., 2020). Recently limited but increasing evidence has shown that the exposure of PM2.5 is related to the adverse effects of the human central nervous system (CNS), from development impairment, synaptic dysfunction, and promoting neurodegeneration (Bhatt et al., 2015; Wei et al., 2016). Growing researches have been focusing on the toxicity of PM2.5 to neurogenesis as fine PM can enter alveoli directly and can penetrate the blood–gas and placental barrier, thus posing potential risks to the fetus, which seems to exert an adverse effect on brain maturation in a critical period of development, with changes in the specific functional domain (Onoda et al., 2017; Zhang et al., 2018).

Very recently, there has been an upsurge of interest in the detrimental effects of PM2.5 on eyes (Chua et al., 2019; Gutiérrez et al., 2019; Yang et al., 2019). The eye is one of several organs that are constantly and directly exposed to the external environment (Zhu et al., 2019). Several epidemiological investigations have demonstrated that people with short- or long-term exposure to PM2.5 may cause dry eye, keratitis, and conjunctivitis accompanied by ocular symptoms and signs (irritation, redness, itchiness, tearing, burning, etc.) (Chang et al., 2012; Fu et al., 2017; Gutiérrez et al., 2019; Lu et al., 2019; Mo et al., 2019). It demonstrated that the possible mechanisms lie in the proinflammatory response and cytotoxicity through DNA damage and oxidative stress caused by PM2.5 exposure (Chua et al., 2019; Tang et al., 2019). Nevertheless, most works described observations of the biological and toxicological influences of PM2.5 on the ocular surface through direct contact; fine specific matter can also affect the retina through the blood–retina barrier (Hong et al., 2016; Lee et al., 2020; Lyu et al., 2020).

The retina, an embryologic extension of the central nervous system, is extremely sensitive and vulnerable to environmental insults (Fox, 2015). Most recently, a few clinical studies have shown that long-term exposure to PM2.5 was associated with retinal arteriolar narrowing and venular widening (Adar et al., 2010; Louwies et al., 2016). Moreover, Chua et al. (2019) showed that participants resident in areas of higher levels of PM2.5 absorbance were associated with apparently adverse retinal structural features, including thinner retinal nerve fiber layer, inner nuclear layer, and outer plexiform layer + outer nuclear layers. Kim et al. (2016) reported that Sprague–Dawley rats exposed to PM decreased retinal thickness, particularly rod/cone cell, inner plexiform, and inner and outer nuclear layers. Although toxicological evidence suggests environmental pollutants exposure associated with retinal development, studies utilizing human-derived model systems are still lacking as a result of ethical limitations (Xu et al., 2015; Lee et al., 2020). Promisingly, previous studies have consistently demonstrated that human embryonic stem cell (hESC)-derived retinal organoids (hEROs) may be capable of recapitulating *in vitro* the morphological and molecular features of the developing human retina, which is believed to provide a more closely associated analog for developmental toxicity assessments of environmental chemicals (Zhang and Ding, 2011; Nakano et al., 2012; Gong et al., 2019; Zou et al., 2019). Additionally, our previous study proved that intermittent high oxygen influenced the formation of neural retinal tissue from hESCs, which simulated closely the early neuroretinal development before angiogenesis and demonstrated the key role of oxygen in the development (Gao et al., 2016). Interestingly, PM2.5 could cross the placenta and fetal blood–brain barrier, causing neurotoxicity of embryonic development (Block and Calderon-Garciduenas, 2009; Perera and Herbstman, 2011; Zhang et al., 2018; Zhao et al., 2020). However, the effects of PM2.5 exposure on retinal development are still unknown.

To the best of our knowledge, there are few studies concerning the influences of PM2.5 on human retinal development using hEROs modeling culture. The present study was to establish a PM2.5 exposure hEROs model and investigate the effects and the potential mechanism of exposure to PM2.5 on retinogenesis.

## Materials and Methods

### hESCs and PM2.5 Preparation

All experiments involving human cells and tissues were carried out following the Tenets of the Declaration of Helsinki and were approved by the Committee on the Ethics of Southwest Hospital, Army Medical University (Chongqing, PR China). Human embryonic stem cell line (H9) was kindly provided by Stem Cell Bank, Chinese Academy of Sciences. This cell line was used in the present study and maintained in CTS Essential 8 Medium (Gibco) without feeders (Gu et al., 2017). The SRM 1648a standard PM2.5 was purchased from NITS (The National Institute of Standards and Technology, Gaithersburg, MD, United States). According to the certificate of analysis of the SRM 1648a, PM2.5 constitutes the largest part of the elements, including Zinc (Zn), Titanium (Ti), Sodium (Na), Chlorine (Cl), etc. (Love et al., 2014).

### Generation of hESC-Derived Retinal Organoids (hEROs)

The generation of hEROs was according to Kuwahara’s inducing protocol and was with slight modifications of the procedure described in our previous studies (Kuwahara et al., 2015; Gong et al., 2019; Zou et al., 2019). In detail, cloned hESCs were digested into single-cell suspensions in TrypLE Express (Gibco) containing 0.05 mg/ml DNaseI (Roche) and 20 μM Y-27632 (Merck). On day 0, 100 μl of cell suspensions (1.2 × 10^4^ cells) with the retinal differentiation medium were forced aggregation in each well (low-cell-adhesion V-bottom 96-well plates, Sumitomo Bakelite) with 5% CO_2_/37°C environment. The retinal differentiation medium consisted of 45% IMDM (Gibco), 45% F12-Glutamax (Gibco), 450 μM monothioglycerol (Sigma-Aldrich), and 1% Chemically Defined Lipid Concentrate (Gibco) supplemented with 10% knockout serum replacement (KSR, Gibco) and 20 μM Y-27632. On day 6, the medium was completely exchanged for retinal differentiation medium supplemented with 1.5 nM bone morphogenetic protein 4 (BMP4, Peprotech). Since then, half of the medium was replaced every 3 days. On day 18, hEROs were transferred into low-cell-adhesion dishes (Greiner) with long-term culture medium containing Dulbecco’s modified Eagle’s medium (DMEM)/F12-Glutamax (Gibco) with 1% N2 supplement (Gibco), 10% fetal bovine serum (FBS, Gibco), 0.5 μM RA (Sigma) and 0.1 mM taurine (Sigma).

### PM2.5 Exposure and Organoid Growth Assessment

PM2.5 (2 g, Urban Particulate Matter, NIST SRM 1648a) was dissolved in phosphate-buffered saline (PBS) at a stock concentration of 10 mg/ml stored at 4°C. Immediately prior to this experiment, the PM2.5 suspension was shocked in a cell disruption sonicator for 10 min to minimize the particle aggregation. On day 18, the hEROs were transferred into low-cell-adhesion dishes and then exposed to fresh long-term culture media containing a series of concentrations of PM2.5, which ranged from 0, 25, 50, to 100 μg/ml (0 μg/ml was used as the control group). The hEROs were treated with PM2.5 for 1, 2, and 3 weeks, respectively (means 3D culture for 25, 32, and 39 days). To ensure accurate concentration, fresh medium, and corresponding concentration PM2.5 were directly added on the first day of each week.

After PM2.5 exposure, the hEROs were observed and photographed using a phase-contrast microscope (Leica DMI3000 B). The neural retina (NR) structures and non-NR structures were identified and counted for 1 week after treatment. The shadow area of the entire hEROs and the thickness of NR were measured by ImageJ-win32 software (NIH, Bethesda, MD, United States); the thickness measurement avoids measuring the ciliary margin of NR, including four treatment groups and three processing time points.

### Immunofluorescence Staining

To identify the characteristics of the NR of hEROs, three hEROs were randomly picked from each of the four groups for 1 and 3 weeks after PM2.5 treatment, respectively. The hEROs were fixed with 4% paraformaldehyde (PFA) for 30 min at 4°C and then transferred to 30% sucrose solution for dehydration at 4°C overnight. A total of nine hEROs were picked from each group at each time point and three independent experiments were performed. After embedding in Optimal Cutting Temperature (OCT, Sakura FineTek, Torrance, CA, United States), the hEROs were cut into 10-μm-thick sections and then attached to glass slides, and the sections were stored at −20°C temperature before immunostaining. Immunofluorescence staining of sections was performed as we described previously (Gao et al., 2016). Briefly, the sections were rinsed in 0.01 M PBS. After treatment with 0.5% Triton X-100 for 10 min and blocking with 3% bovine serum albumin (BSA) for 60 min at room temperature, the sections were incubated with the primary antibodies with 0.3% Triton X-100 and 3% BSA at 4°C overnight. The following primary antibodies and dilutions had been used in the experiments: mouse anti-Chx10 (Santa Cruz, sc-374151, 1:200), rabbit anti-Chx10 (Sigma-Aldrich, HPA003436, 1:1000), mouse anti-Pax6 (Abcam, ab5790, 1:500), mouse anti-Rax (Santa Cruz, sc-271889, 1:200), rabbit anti-Ki67 (Abcam, ab66155, 1:500), rabbit anti-Cleaved caspase3 (CTS, #9661, 1:500), mouse anti-P27 (Santa Cruz, sc-1641, 1:200), mouse anti-BRN3 (Santa Cruz, sc-8429, 1:200), rabbit anti-HuCD (Abcam, ab184267, 1:400), mouse anti-CRX (Abnova, H00001406-M02, 1:400), rabbit anti-ZO-1 (Invitrogen, 402200, 1:500), and mouse anti RPE65 (Abcam, ab78036, 1:500). On the following day, the sections were incubated with secondary antibodies Goat anti-mouse IgG Alexa-Fluor-488 (Life technologies, A11001, 1:400), Goat anti-rabbit IgG Alexa-Fluor-568 (Life technologies, A11001, 1:800), and Goat anti-mouse IgG Alexa-Fluor-647 (Life technologies, A21236, 1:800) applied for 2 h at room temperature. After nuclear counterstaining with DAPI, the sections were examined and photographed using the confocal laser scanning microscope (Leica SP5).

### TUNEL Assay

The cell apoptosis was determined according to the TUNEL (TdT-mediated dUTP-X nick end labeling) assay kit manual (*In Situ* Cell Death Detection Kit, Roche, Basel, Switzerland). After treatment with PM2.5 at different concentrations and periods, the sections of hEROs were treated before the primary antibody incubated as described above. For TUNEL staining, buffers 1 and 2 were mixed at a ratio of 1:9 according to the manufacturer’s instructions and then diluted at 1:3 with PBS, and finally incubated with the TUNEL reaction mixture at 37°C for 30 min in a dark and humidified box. Nuclei were counterstained with DAPI for 5 min. The sections were visualized using a Leica SP-5 confocal microscope. The number of NR-TUNEL-positive cells was counted randomly.

### Immunoreactive Cell Counting

Assessment of cell apoptosis with TUNEL and cell proliferation with Ki67 has performed at least three comparable hERO sections across the NR in the four treatment groups after PM2.5 exposure for 1 and 3 weeks. In addition, the hERO sections were labeled with the retinal progenitor marker Chx10 and the CDK inhibitors p27 for the control group and 100 μg/ml PM2.5 exposure group, with exposure for 3 weeks. The number of cells was counted by using Adobe Photoshop CS5 software (NIH, Bethesda, MD, United States). In detail, high-resolution images of NR were acquired, and the number of positive cells and the overall DAPI cells were counted manually among the three NR regions as large as possible, and positive percentages were counted at last.

### Transcriptome Analysis and Real Time-PCR

For a more comprehensive analysis of PM2.5-treated hEROs, the expression profile of the PM2.5 high-concentration group (100 μg/ml) and the control group was examined for 3 weeks after PM2.5 treatment (the high-dose group was defined as PM2.5-100 μg/ml, PM2.5-0 μg/ml was defined as the control group); three independent biological replicates are set for each group. Total RNA was extracted with Trizol (Invitrogen, Carlsbad, CA, United States) according to manual instruction and was qualified and quantified using a NanoDrop and Agilent 2100 bioanalyzer (Thermo Fisher Scientific, MA, United States). Subsequently, Oligo (dT)-attached magnetic beads were used to purified mRNA. The cDNA fragments were obtained from the previous step followed by PCR amplification. The quality control of PCR product was performed on an Agilent Technologies 2100 bioanalyzer.

Gene expression levels were estimated using the FPKM value (fragments per kilobase of transcript per million fragments mapped). The differentially expressed genes (DEGs) between PM2.5-100 μg/ml and control groups were analyzed with the DEG-seq method. Fold changes ≥ 1 and adjusted *P* ≤ 0.01 were defined as indicative of DEGs. The significantly differential genes between PM2.5-100 μg/ml and control group were acquired by high-throughput gene sequencing analysis, and then the DEGs were analyzed by Gene Ontology (GO) and Kyoto Encyclopedia of Genes and Genomes (KEGG) pathway. According to the interaction network between the pathways in the KEGG database, the interactions of the obtained significantly differential genes enriched in the KEGG pathway were extracted from the database. Then, these interactive relationship files were imported into Cytoscape 3.5.0 software and drawn into a KEGG path interaction network diagram. RT-PCR expression validations were conducted using the CFX96 Real-Time PCR System (BioRad, Hercules, CA, United States). The primers were produced by Sangon Biotech (Shanghai, China), and the primers used are listed in Supplementary Table S1.

### Statistical Analysis

All data were using the Statistical Product and Service Solutions software V17.0 (SPSS, Chicago, IL, United States). For comparison between multiple groups, data were analyzed by one-way ANOVA followed by Fisher’s protected least-significant difference *post-hoc* tests. For comparison between two groups, the unpaired Student’s *t* test was performed. All data were expressed as the mean ± standard error. The significance level was set at 0.05, and the statistical figure was drawn using GraphPad Prism 6.01.

## Results

### Identification of hEROs-Derived NR

**FIGURE 1:**
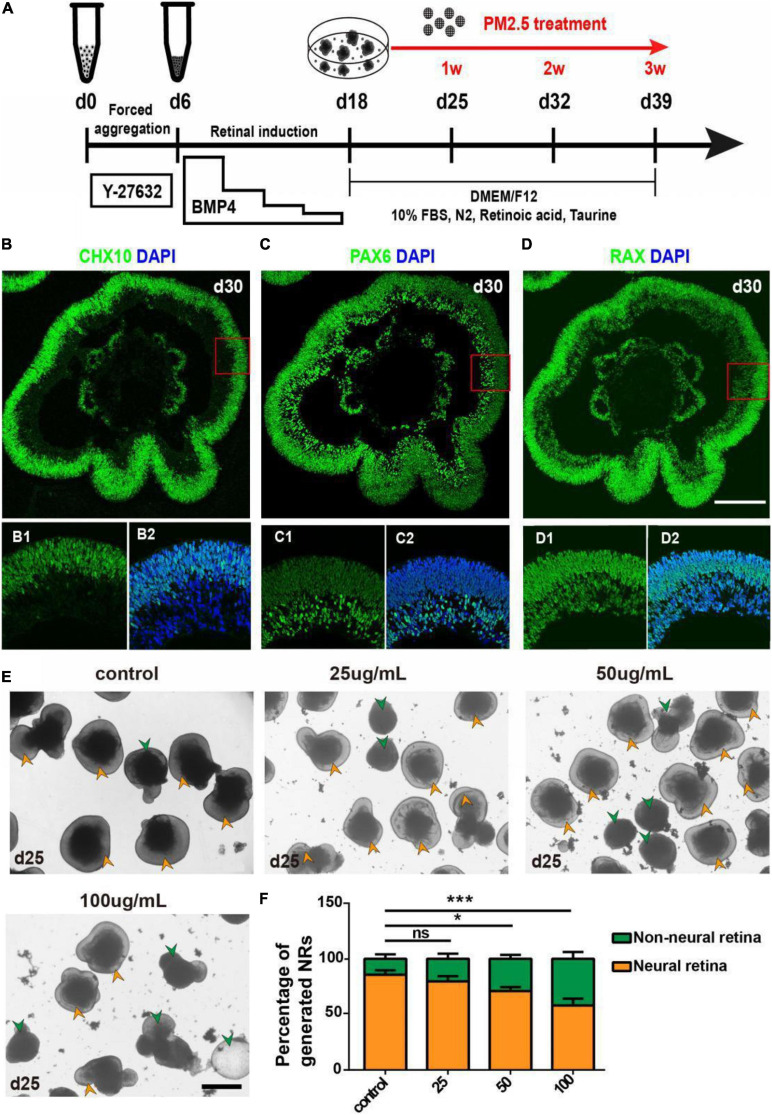
Formation and identification of three-dimensionally induced hEROs and effects of PM2.5 treatment on the formation rate of hERO-NRs. **(A)** Schematic diagram of 3D-induced hEROs formation and PM2.5 treatment. **(B–D)** Identification of RPC markers CHX10, PAX6, and RAX in hEROs at day 30, respectively. Scale bar = 50 μm. Green, FITC; Blue, DAPI. **(E)** Bright-field view showing the formation of hERO-NRs after treatment with PM2.5 at concentrations of control (0), 25, 50, and 100 μg/ml for 1 week, respectively. Yellow and green arrows are pointing at the representative of NR and non-NR at the edges of the hEROs, respectively. Scale bar = 200 μm. **(F)** Statistical analysis of the number of hERO-NRs in different PM2.5 concentration (control vs. 25 μg/ml vs. 50 μg/ml vs. 100 μg/ml, respectively. Treatment time was 1 week. *n* = 3 independent experiments per group; each group contains 72 hEROs. NS, no significant difference, *P < 0.05, ***P < 0.001).

To begin with, we used hEROs to model early retinal development. The generation of hEROs was conducted as described in our previous studies (Kuwahara et al., 2015; Gong et al., 2019; Zou et al., 2019). PM2.5 treatment was set at day 18, the time NRs were generated within hEROs initially, then continued to observe the formation and differentiation of NR for 1–3 weeks (Figure 1A). On day 30, most of the NR generated under this condition was positive for Chx10, Rax (NR progenitor marker), and Pax6 (early retina marker; Figures 1B–D). Immunofluorescence results showed that Chx10-positive cells were mainly localized at the apical side of NR, while Pax6-positive cells were mainly distributed at the basal side of NR, and Rax positive cells were distributed in both the apical and basal sides (Figures 1B1–D2). Together, we successfully repeated retinal development with the inducing method in this study.

### PM2.5 Affected the Formation of hEROs-NR

The numbers of NR and Non-NR were counted after PM2.5 exposure with different concentrations (0, 25, 50, and 100 μg/ml) for 1 week(day 25), then the ratio of generated NRs was calculated. Green arrows that indicated the smaller and poorer refraction hEROs were defined as Non-NR and yellow arrows showed the typical NR with protruding neuroectodermal epithelial layer (Figures 1E,F). The formation rate of hERO-NRs reached 90% at day 25 without PM2.5 treatment (control group). The NR formation rates decreased significantly after exposure to PM2.5 at 50 μg/ml (*P* = 0.0138) and 100 μg/ml (*P* = 0.0003) concentrations for 1-week exposure, which suggested that the formation rate of hERO-NRs was affected by PM2.5 in a dosage-dependent manner.

### PM2.5 Affected the Area of hEROs and the Thickness of hERO-NRs

**FIGURE 2:**
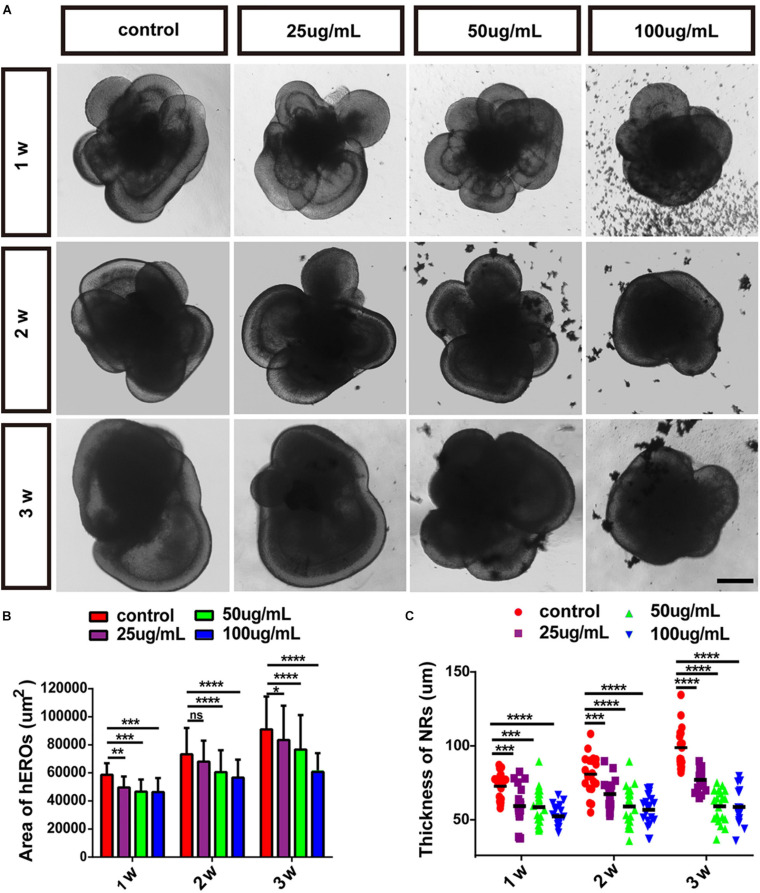
The effects of PM2.5 on the area of hEROs and the thickness of hERO-NRs. **(A)** Bright-field images of hEROs after exposure to four concentrations, group of PM2.5 continuous processing three periods at 1, 2, and 3 weeks. Scale bar = 100 μm. **(B)** The area of the hEROs in each group was calculated and statistically analyzed. **(C)** The thickness of the neural retina in each group was calculated and statistically analyzed (control vs. 25 μg/ml vs. 50 μg/ml vs. 100 μg/ml) in PM2.5-treated hEROs after 1, 2, and 3 weeks, respectively. *n* = 3 independent experiments per group; each group contains 40 hEROs (*P < 0.05, **P < 0.01, ***P < 0.001, ****P < 0.0001).

We chose the NR-generated hEROs in the control group and PM2.5 treatment group for further analysis. The area of hEROs and the thickness of hERO-NR decreased gradually with increasing PM2.5 concentration at all three time points. Compared with the control group (58,652 ± 4,609 μm^2^), the area of hEROs with 25 μg/ml PM2.5 (49,638 ± 3,770 μm^2^, *P* = 0.0072), 50 μg/ml PM2.5 (46,609 ± 5,053 μm^2^, *P* = 0.0002), and 100 μg/ml PM2.5 (46,362 ± 5,906 μm^2^, *P* = 0.0001) exposure was significantly decreased after 1 week. After PM2.5 exposure for 2 weeks, the area of hEROs with 25, 50, and 100 μg/ml PM2.5 exposure was lower than that of the control group (68,042 ± 9,625 μm^2^, *P* = 0.1929; 60,596 ± 8,359 μm^2^, *P* < 0.0001; 56,668 ± 5,207 μm^2^, *P* < 0.0001; 73,261 ± 11,842 μm^2^, respectively). After PM2.5 exposure for 3 weeks, the area of hEROs with 25, 50, and 100 μg/ml PM2.5 exposure was lower than that of the control group (83,468 ± 13,035 μm^2^, *P* = 0.0289; 76,671 ± 11,180 μm^2^, *P* < 0.0001; 60,701 ± 6,656 μm^2^, *P* < 0.0001; 91,089 ± 11,071 μm^2^, respectively) (Figures 2A,B).

Compared with the control group (82.32 ± 8.00 μm), the thickness of hERO-NR with 25 μg/ml PM2.5 (70.21 ± 11.36 μm, *P* = 0.0005), 50 μg/ml PM2.5 (69.64 ± 10.84 μm, *P* = 0.0003), and 100 μg/ml PM2.5 (64.06 ± 6.28 μm, *P* < 0.0001) exposure was significantly decreased. After PM2.5 exposure for 2 weeks, the thickness of hERO-NR with 25, 50, and 100 μg/ml PM2.5 exposure was lower than that of the control group (77.57 ± 8.53 μm, *P* = 0.0006; 70.06 ± 11.80 μm, *P* < 0.0001; 68.11 ± 9.18 μm, *P* < 0.0001; 89.59 ± 11.60 μm, respectively). After PM2.5 exposure for 3 weeks, the area of hEROs with 25, 50, and 100 μg/ml PM2.5 exposure was lower than that of the control group (86.21 ± 5.87 μm, *P* < 0.0001; 70.14 ± 9.45 μm, *P* < 0.0001; 69.71 ± 9.93 μm, *P* < 0.0001; 105.83 ± 11.90 μm, respectively; Figure 2C). These results suggested that the growth of hEROs and hERO-NR was suppressed by PM2.5 in a dosage-dependent manner, and the most significant effect was demonstrated at a concentration of 100 μg/ml for 3 weeks (day 39).

### PM2.5 Affected the Proliferation and Apoptosis of Cells in the hERO-NRs

**FIGURE 3:**
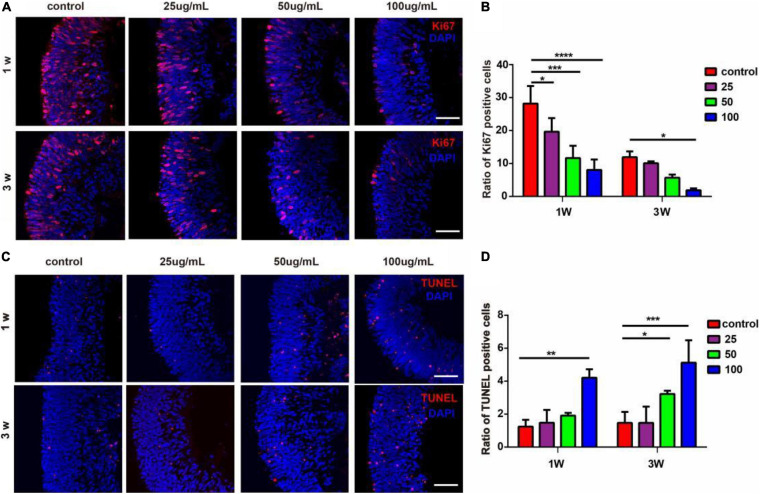
The effects of PM2.5 on the proliferation and apoptosis of hERO-NRs. **(A)** Ki67-immunoreactive proliferating cells within the hERO-NRs after 1-week and 3-week treatment with 0, 25, 50, and 100 μg/ml PM2.5 concentrations. Scale bar = 50 μm. **(B)** The statistical analysis of Ki67-positive cell ratio of the hERO-NRs in four groups. **(C)** TUNEL-immunoreactive apoptosis cells within the NR after 1 and 3 weeks of treatment with different PM2.5 concentrations. Scale bar = 50 μm. **(D)** The statistical analysis of TUNEL-positive cell ratio of the hERO-NRs in four groups (control vs. 25 μg/ml vs. 50 μg/ml vs. 100 μg/ml PM2.5-treated hEROs at 1 and 3 weeks, respectively). *n* = 3 independent experiments per group; each group contains 30 hEROs (*P < 0.05, **P < 0.01, ***P < 0.001, ****P < 0.0001).

To assess the impact of PM2.5 on cell proliferation and apoptosis of hERO-NRs, we performed Ki67 and TUNEL staining. It demonstrated that all concentrations of PM2.5 exposure for 1 week decreased the ratio of Ki67-positive cells significantly compared with the control group (Figures 3A,B). PM2.5 at the dosage of 25 and 50 μg/ml showed no significant effect on the percentage of Ki67-positive cells when exposed for 3 weeks, while it markedly decreased the ratio of Ki67-positive cells with 100 μg/ml.

Apoptosis was increased as the concentration of PM2.5 increased. The ratio of TUNEL-positive cells was significantly increased with exposure of 100 μg/ml PM2.5 for 1 week and 50 μg/ml and 100 μg/ml PM2.5 for 3 weeks, compared with the control group (Figures 3C,D). PM2.5 at the dosage of 25 μg/ml showed no significant effect on the ratio of TUNEL-positive cells. These results indicated that PM2.5 suppressed hERO-NR cell proliferation and promoted hERO-NR cell apoptosis, and these effects were time and dosage dependencies.

### Influences of PM2.5 Exposure on the Cellular Characteristics of hERO-NRs

**FIGURE 4:**
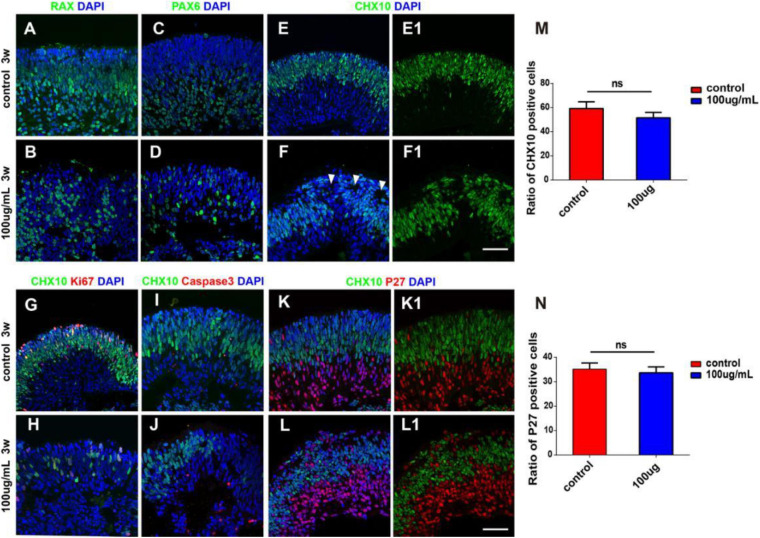
The characterization of hERO-NRs with 100 μg/ml PM2.5 exposure. **(A–F)** Immunostaining analysis of RPC-related markers in the hERO-NRs. The RPC marker Rax-positive cells (green), Chx10-positive cells (green), and Pax6-positive cells (green) between control and PM2.5-100 μg/ml treatment after 3 weeks. White arrows of **(F)** are pointing at typical neural rosettes in several areas of hERO-NRs. **(E1,F1)** show **(E,F)** without DAPI, respectively. **(G–J)** Non-co-expression between the apoptosis marker caspase3 (red) and TUNEL with the proliferation marker Ki67 (red) within the missing Chx10 (green) cells appeared. **(K,L)** Most missing Chx10-positive cells (green) express the cell cycle exit marker P27 (red). **(K1,L1)** show **(K,L)** without DAPI, respectively. Scale bar = 50 μm. **(M)** Statistical analysis of Chx10-positive cells. **(N)** Statistical analysis of P27-positive cells (NS, no significant difference).

At the early stage of retinal development, hERO-NRs are mainly composed of retinal progenitors, which will gradually exit the cell cycle and begin to differentiate. We further analyzed the influence of 100 μg/ml PM2.5 exposure on the retinal progenitors of hERO-NRs. After PM2.5 exposure, we found that the distribution of Chx10-, Rax-, and Pax6-positive cells was disordered and some cells between them were negative of retinal progenitor markers (Figures 4A–F). More importantly, typical neural rosettes were found in several areas of hERO-NRs in the 100 μg/ml PM2.5-exposed group, yet hardly observed in the control group and other low-concentration groups (Figure 4F, white arrows). We also found that the proliferative cells (Ki67 positive) decreased in the Chx10^+^ cell region (apical side) and the apoptotic cells (Caspase3 positive) increased in the Chx10^–^ cell region (basal side; Figures 4G–J). Although the distribution of progenitor markers appeared abnormal as described above, the difference in the proportion of Chx10^+^ cells between the control group and the PM2.5 treatment group was not statistically significant (Figure 4M). Furthermore, it demonstrated that cycle-dependent kinase inhibitor p27 (a cell cycle inhibitor)-positive cells, which indicated cell differentiation, mainly distributed in the Chx10^+^ cell region (Figures 4K,L), but they did not show a significant difference between the PM2.5-exposed group and control group (Figure 4N). Together, we showed that the ratio of differentiated retinal cells and retinal progenitors was not significantly different between the PM2.5 treatment group and control group, indicating that PM2.5 exposure mainly leads to retinal cell dislocation but not the promotion of cell differentiation.

### PM2.5 Affected the Differentiation of hERO-NRs

**FIGURE 5:**
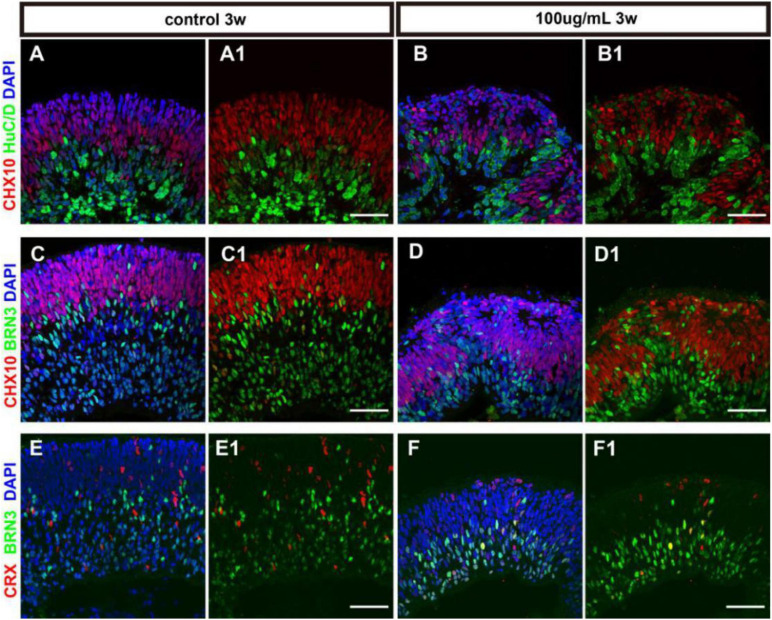
The cell differentiation of hERO-NRs with 100 μg/ml PM2.5 exposure. **(A–D)** Immunostaining analysis of retinal differentiation-related markers in the hERO-NRs (after 3 weeks, 100 μg/ml PM2.5 treatment). **(A,B)** Co-tagging of Chx10 and HuCD at control group and PM2.5-100 μg/ml group. **(C,D)** Co-tagging of Chx10 and BRN3 in the control group and PM2.5-100 μg/ml group. **(A1–D1)** shows **(A–D)** without DAPI, respectively. **(E,F)** Immunostaining analysis of CRX and BRN3 in the hERO-NRs (after 3 weeks, 100 μg/ml PM2.5 treatment). **(E1,F1)** show **(E,F)** without DAPI, respectively. Scale bar = 50 μm.

Retinal progenitors are capable of differentiating into different retinal cells including photoreceptors and other retinal neurons. The differentiation of cell subtypes on hERO-NRs revealed that in the presence of 100 μg/ml PM2.5, HuC/D (interneurons marker)- and Brn3 (ganglion cell marker)-positive cells appeared on both apical and basal side, which mainly expressed at the basal side with regular stratification in the control group (Figures 5A–D). The distribution of Crx (photoreceptor precursor cells marker)-positive cells in the 100 μg/ml PM2.5 exposed group seemed to be consistent with the control group (Figures 5E,F), and the ratio was not significantly different (data not shown). In addition, we noticed that an abundance of Brn3-positive ganglion cells migrated into the Chx10^+^ cell region in hERO-NRs after exposure to PM2.5, and the number of migrated Brn3-positive cells in the Chx10^+^ cell region increased significantly in the PM2.5 group. However, the number of Brn3-positive cells showed no significant difference between the PM2.5-exposed group and the control group in the whole hERO-NRs (Supplementary Figure S1). These data suggested that PM2.5 treatment did not produce significant effects on the differentiation of the retinal cells in hERO-NRs but had a significant influence on migration and dislocation of the ganglion cells.

### Transcriptome Analysis of hEROs After PM2.5 Treatment

**FIGURE 6:**
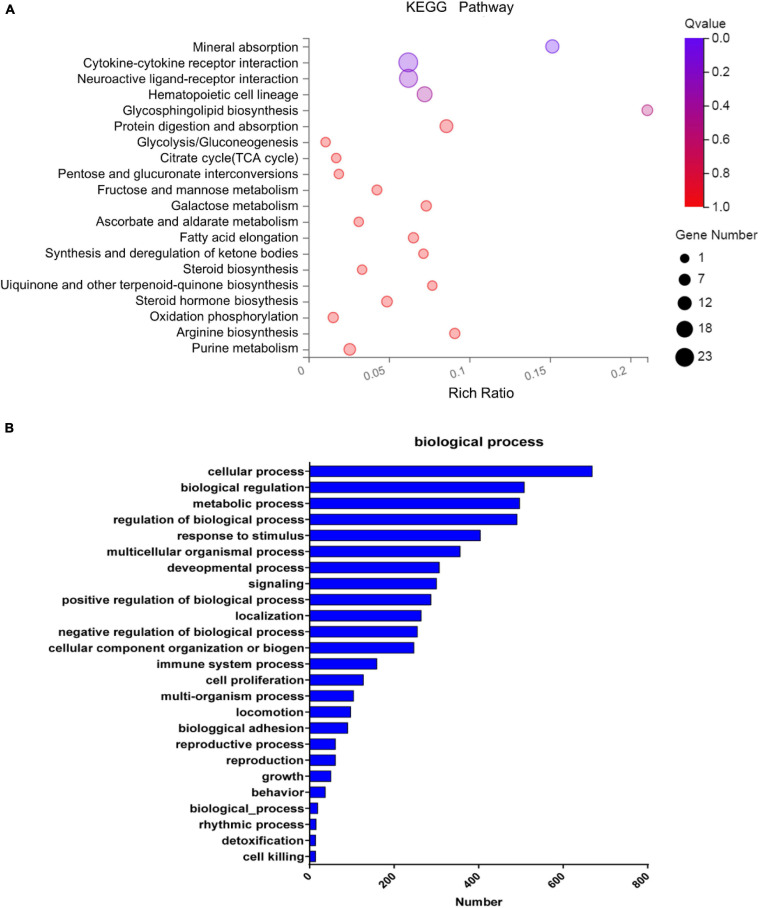
The enrichment analysis of differentially expressed genes. **(A)** The KEGG pathway enrichment of differentially expressed genes. Rich factor and *Q* value indicate the intensiveness of enrichment. The scale of plots stands for the gene numbers. **(B)** Classification of the identified differentially expressed genes into the biological process.

Gene expression profile of hERO-NRs treated with 100 μg/ml PM2.5 for 3 weeks will help us to understand the toxic mechanism more clearly. Therefore, mRNA sequencing was performed in hERO-NRs. A total of more than 6.8 billion clean reads were generated from all six cDNA libraries using the BGISEQ-500 platform. The gene expression with fold changes ≥ 2 and adjusted *P* ≤ 0.01 was identified as a significant difference (Supplementary Tables S2, S3). The gene ratio distribution of fold change was shown in Supplementary Figure S2. The results of mRNA sequencing showed that a total of 2,587 significantly expressed genes were identified. Among them, 2,002 genes were expressed at significantly higher levels and 587 genes were expressed at significantly lower levels in the PM2.5 group than the control group (Figure 6A). Meanwhile, the Gene Set Enrichment Analysis (GSEA) method was applied to show the concordant differences between two biological states (see Supplementary Figure S3). The heatmap of relative expression levels of differently expressed genes (DEGs) of hEROs with 100 μg/ml PM2.5 exposure is shown in Supplementary Figure S4.

Pathway enrichment based on the KEGG database generated a scatterplot of pathway terms of DEGs, as shown in Figure 6A. The top 20 significant pathways include mineral absorption, cytokine–cytokine receptor interaction, neuroactive ligand–receptor interaction, hematopoietic cell lineage, etc. Based on GO analysis, the identified DEGs were classified into biological processes as shown in Figure 6B, including cellular process, biological regulation, metabolic process, regulation of the biological process, response to the stimulus, multicellular organismal process, cell proliferation, etc. According to GO analysis, the identified DEGs were also classified into molecular functions and cellular components, as shown in Supplementary Figure S5.

**FIGURE 7:**
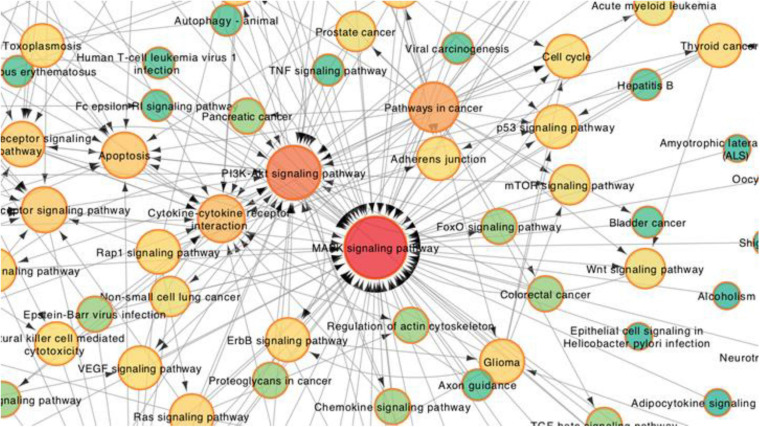
Partial enlargement of the pathway-act network analysis between PM2.5-100 μg/ml treatment with the control group of the hEROs at 3 weeks. Each node represents a signaling pathway. Arrows represent interactive relationships between two signaling pathways. The size and color of the circle represent the significance of the difference between the groups in the KEGG pathway. The circular red shades represent the clustering coefficients. A darker color indicates more strongly associated pathways. The direction of the arrow represents the upstream and downstream relationship of the path.

**FIGURE 8:**
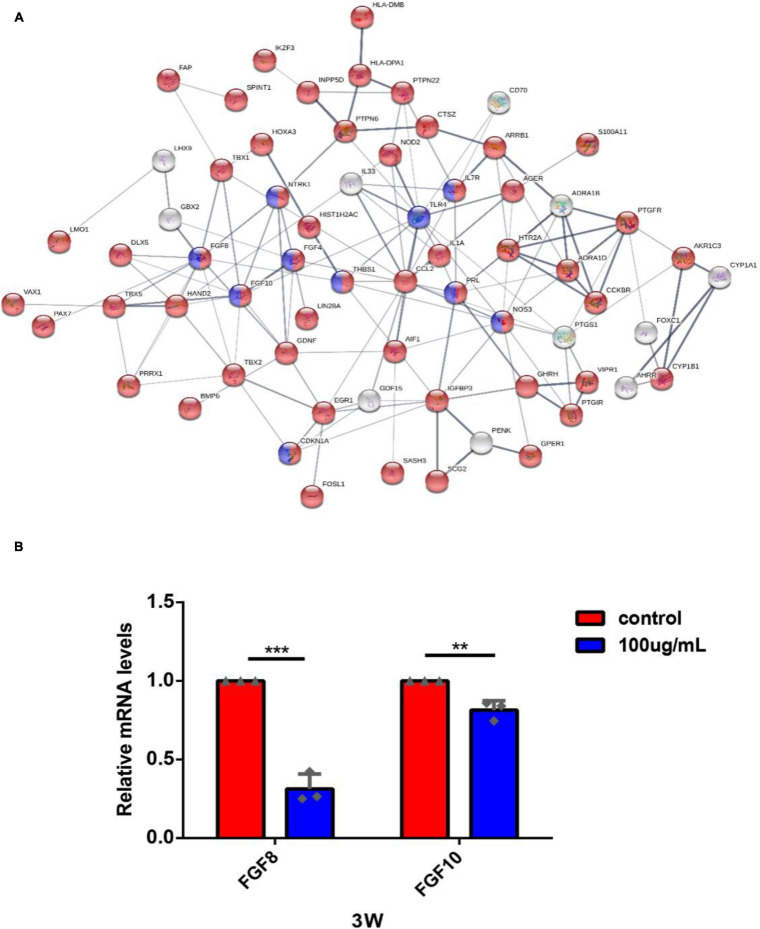
The gene-act network was constructed based on the interactions among cell proliferation-related genes in the STRING database and RT-PCR validation. **(A)** Each circle represents a gene. Lines represent interactive relationships between two signaling pathways. The disconnected nodes in the network were hidden. Red color means DEGs related to the biological process of regulation of cell population proliferation; blue color means DEGs related to the KEGG pathway of PI3K/Akt signaling pathway. **(B)** The expression of FGF8 and FGF10 of hEROs with PM2.5 exposure was detected by RT-PCR (**P < 0.01, ***P < 0.001).

To further explore the core pathways underlying the effects of PM2.5 in hEROs-NRs, we constructed a pathway interaction network map based on the relationships among pathways in the KEGG database. The core part of the entire network interaction diagram was shown separately (Figure 7). After considering the significance of the identified pathway differences and the interaction network, we found that the following three KEGG pathways were significant in the interaction network: the MAPK signaling pathway, the PI3K/Akt signaling pathway, and cytokine–cytokine receptor interaction. Additionally, the mTOR signaling pathway, p53 signaling pathway, apoptosis pathway, Wnt signaling pathway, etc. were also shown to be significantly enriched in the network. The darker color indicated that more genes were associated. The direction of the arrow represents the upstream and downstream relationship of the path. According to the cellular biological process based on GO analysis, we focused on cell proliferation. A total of 127 correlated genes were submitted to the Search Tool for the Retrieval of Interacting Genes (STRING)^1^ database to construct an interaction network (Figure 8A). Among genes in interaction, the network was associated with the biological process of regulation of cell population proliferation and the KEGG pathway of PI3K/Akt signaling pathway.

Among the interacting genes (Figure 8A), we identified two important genes, FGF8 and FGF10, which are related to cell proliferation and retinogenesis (Fuhrmann, 2010; Rash et al., 2011). The RT-PCR was performed to verify that the expression of FGF8 and FGF10 in the PM2.5-exposed groups was lower than the control (0.31 ± 0.08, *P* = 0.002; 0.81 ± 0.05, *P* = 0.0059, respectively). In addition, we also found that some cytokines and their receptors associated with immune function, including TLR4, IL-7R, IL-1A, and CCL2, were upregulated (Supplementary Table S2) in the PM2.5 group, which was verified by RT-PCR (3.13 ± 0.34, *P* = 0.0056; 5.36 ± 0.35, *P* = 0.0002; 2.40 ± 0.10, *P* = 0.0001; 2.88 ± 0.26, *P* = 0.0019, respectively) (Supplementary Figure S6). Given the importance of telomerase activity in stem cell proliferation/differentiation, and linked to inflammatory response Rap1/NFkb activity (Zhang et al., 2015), there were no significant differences between the expression of telomerase activity gene, such as Rap1 in the PM2.5 group and control group in our transcriptome data. In addition, our results showed that the telomerase activity and inflammatory pathway Rap1/NFKb activity was not a key pathway in KEGG analysis and the pathway interaction network map (Figures 6, 7).

**FIGURE 9:**
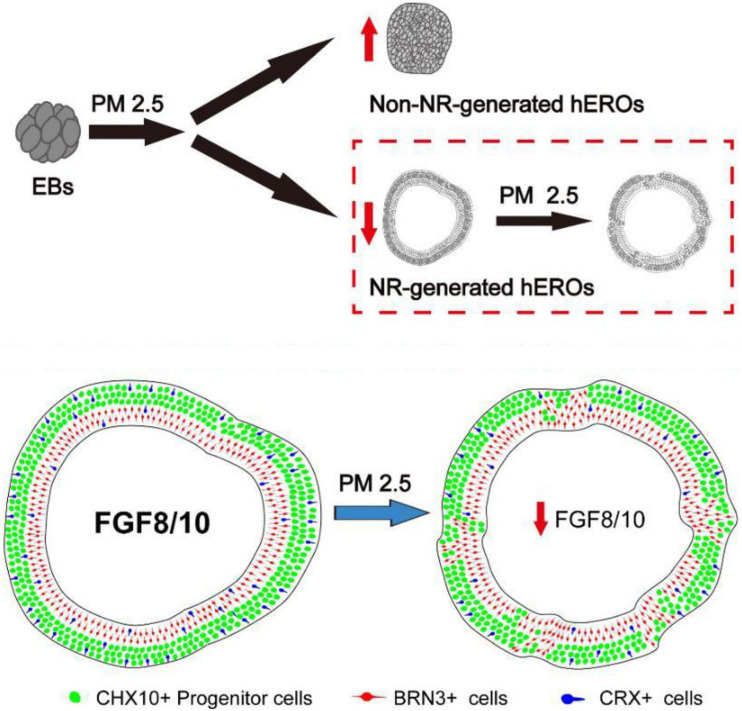
Summary of effects of particulate matter (PM2.5) on human retinal development in hESC-derived retinal organoids. PM2.5 exposure on PM2.5 might inhibit the early neural retinal formation and subsequent cell subtypes dislocation through downregulation of FGF8 and FGF10.

As shown in Figure 8B, the RT-PCR results were essentially consistent with transcriptome results. Those transcriptional results indicated that PM2.5 might inhibit the early neural retinal formation and subsequent cell subtype dislocation through downregulation of FGF8 and FGF10 (Figure 9).

## Discussion

Due to strict ethical restrictions and heterogeneity of animal tissues, research on the harmful effects of air pollutants on human eye development has been greatly limited. In this study, we applied hESC-derived retinal organoids to evaluate the developmental toxicity of PM2.5. To the best of our knowledge, this is the first time to study the relationship between PM2.5 exposure and human retinal development.

After exposure to PM2.5, adult mice showed impaired learning and memory abilities, suggesting that PM can adversely affect the synaptic structure and cognitive competence of the CNS by activating reactive oxygen species and pro-inflammatory cytokines (Sagai and Tin Win-Shwe, 2015). In addition, there are several reports that showed that PM2.5 exposure is related to the development of retinal degeneration, such as glaucoma and retinal morphology changes. Chua’s work showed that increased exposure to PM2.5 is associated with self-reported glaucoma and poor structural characteristics of the disease (Chua et al., 2019). Another research showed that participants who lived in areas with higher levels of PM2.5 absorbance were more likely to have retinal morphology changes (Chua et al., 2020). As for retinal development, there is no direct evidence about PM2.5 exposure with developmental eye impairments including microphthalmia. Notably, it was reported that PM2.5 could cross the placenta barrier and enter the fetus vessel, causing CNS damage (Woodward et al., 2015; Zhang et al., 2018; Zhao et al., 2020). However, the research on the effect and mechanism of exposure to PM2.5 on the development of CNS is still limited, and even less on the development of the retina.

It is well-known that the specification of hESC-derived 3D retinal organoids recapitulates development in the human retina. In this study, we examined the developmental dynamics of cell subtype specification in retinal organoids. Consistently, our previous studies also showed that the markers of Chx10, Rax, and Pax6 were observed in hEROs at day 30 (Zou et al., 2019). In this study, toxic influences of PM2.5 exposure on retinal development were determined by the physical and biological evaluations of hERO-NR. Our result showed that the formation rate of hERO-NR was severely affected after treatment with PM2.5, particularly with 50 and 100 μg/ml PM2.5. The decreased ratio of generated NRs was negatively associated with PM2.5 levels. Moreover, the reduced area and the poor refractive index of hEROs were associated with PM2.5 exposure dose-dependently. The abnormal morphologies of hEROs, possibly due to PM2.5, affected cellular biological process. Subsequently, the thickness of hERO-NR was found to be negatively associated with increasing PM2.5 concentration in our study, which could be an indicator to assess the cell proliferation and differentiation of hEROs. The ratio of TUNEL-positive cells showed that the increased apoptosis cells were positively associated with PM2.5 at the 100 μg/ml level. This result was consistent with lower Ki67-immunoreactive proliferating cells. The lower ratio of Ki67-positive cells was also positively associated with PM2.5 at the 100 μg/ml level. These results indicated that PM2.5 with a higher level of 100 μg/ml suppressed hERO-NR cell proliferation, while promoting cell apoptosis. Consistently, Fu et al. (2017) observed decreased cell viability and proliferation, as well as increased apoptosis in human corneal epithelial cells after PM2.5 exposure for 24 h. Tan et al. (2018) also showed PM2.5-induced apoptosis in the corneal surficial and basal epithelium, abnormal proliferation, and differentiation of the ocular surface. Additionally, the retina is a highly ordered tissue with layered cell subtypes. We observed that PM2.5 exposure resulted in the appearance of neural rosette-like gaps, which confirmed that PM2.5 exposure induced the disordered structure (Zheng et al., 2009). Therefore, our results suggested that PM2.5 exposure could influence the cell proliferation and apoptosis of hEROs.

Generally, retinal ganglion cells, interneurons cells, and photoreceptor precursor cells will be generated on day 39 with our method. Therefore, these cells were used to identify the specific structure of the hERO-NRs upon PM2.5 exposure. The abnormal cell distributions and structural disorder, particularly apoptosis of retinal ganglion cells, were observed in PM2.5-exposed hERO-NR. It is consistent with Chua’s study, which found a dose–response relationship between higher levels of PM2.5 and thinner macular ganglion cell–inner plexiform layer (GCIPL) (Chua et al., 2019). Glaucoma is an optic neuropathy characterized by degeneration of retinal ganglion cells, and the absence of GCIPL may reflect structural changes related to glaucoma. Recently, Chua’s group also showed that participants resident in areas of higher levels of PM2.5 absorbance were more likely to have thinner retinal nerve fiber layer, inner nuclear layer, and outer plexiform layer and outer nuclear layers, as illustrated by SD-OCT imaging measurement (Chua et al., 2020). Moreover, the results of cellular characteristics of hERO-NRs showed that PM2.5 exposure caused the missing of various specific cell markers, rosette structure, and the entire NR layer to become thinner. Likely, the disordered structure could be caused by cell apoptosis induced by PM2.5. Another possibility was due to the critical signaling pathways being affected by PM2.5, which regulates cytoskeleton dynamics and apical constriction of the NR, leading to different cell migration and even malformation (Miao et al., 2018; Gong et al., 2019). Furthermore, we investigated the underlying toxic mechanisms of PM2.5 through transcriptome analysis of hEROs.

To determine how PM2.5 exposure may drive differential gene expressions associated with abnormal retinal development, we performed RNA-seq on hEROs. Based on the KEGG pathway analysis, mineral absorption, cytokine–cytokine receptor interaction, neuroactive ligand–receptor interaction, hematopoietic cell lineage, etc. were associated with PM2.5 exposure. We speculated that PM2.5 could shape the toxicity mediated by these complicated processes. Furthermore, the entire network interaction of these pathways showed that the MAPK signaling pathway, the PI3K/Akt signaling pathway, the mTOR signaling pathway, p53 signaling pathway, apoptosis pathway, and Wnt signaling pathway were identified as critical pathways involved in the response to PM2.5. Similar to previous studies, the PI3K/Akt pathway activation was caused by PM2.5, which acts as an important role in cardiovascular toxicity (Yang et al., 2019). In contrast, the activation of the mitogen-activated MAPK signaling pathway and PI3K/Akt signaling pathway showed that it protects neurons against various forms of injury (Zhu et al., 2016; Husain et al., 2017). The difference could be caused by a complicated gene interaction network after PM2.5 exposure. Lee et al. (2020) proved that PM2.5 promoted the phosphorylation of MAPKs and induced mitochondrial defects, leading to epithelial–mesenchymal transition of retinal pigment epithelial, which suggested that PM2.5 induced retinal dysfunction. Besides, several pathways in response to PM2.5 are also found to be associated with retinal development, such as the Wnt signaling pathway, mTOR signaling pathway, and cell cycle. Capowski et al. (2016) showed that the Wnt signaling pathway was regulated by the visual system homeobox 2 (VSX2), previously called CHX10, acting to maintain NR identity during optic vesicle patterning. Simultaneously, Yan et al. (2020) found that PM2.5 affected epithelial differentiation of human umbilical cord-derived mesenchymal stem cells by the Wnt/β-catenin pathway. It has been found that the mTOR signaling pathway was associated with many biological processes, including cell growth, proliferation, and apoptosis (Zhang et al., 2019). An experimental study by Love et al. (2014) showed that mTOR signaling mediated the proliferation and differentiation program in retinal progenitor cells. After exposure to PM2.5, the activity of the mTOR/P70S6K1 signaling pathway was inhibited, thus leading to cell cycle arrest (Zhang et al., 2019). These results indicated that PM2.5 exposure potentially affected the proliferation and differentiation of hEROs by complicated gene networks.

Interestingly, the GO terms of identified DEGs are related to cell proliferation. The interactive networks were constructed by cell proliferation-related genes, which were also associated with the PI3K/Akt signaling pathway. Obviously, the fibroblast growth factors (FGFs), particularly FGF8 and FGF10, were significant in the network. FGFs were proved to bind to FGF receptors (FGFRs) and play a key role in retinogenesis, including retinal cell proliferation, migration, and photoreceptor maturation (Forouzanfar et al., 2019). In the developing retina, FGF8 regulated NR formation and triggered retinal progenitors to differentiate, as well as combined with Notch signaling in regulating Müller glia activation and proliferation that contribute to retina growth and regeneration (Wan and Goldman, 2017). In our data, the downregulated FGF8 suggested that the possibility of PM2.5 affected the NR formation and cell subtype dislocation. It was reported that FGF10 was abundantly expressed in the retina (Hsi et al., 2013). The defect in FGF10 led to the development and differentiation of several ocular tissues, and FGF10 modulated extracellular matrix-associated genes that were involved in the development of myopia (Yoshida et al., 2013; Jiang et al., 2019). In our study, the downregulated FGF10 suggested that retinal development was potentially affected by PM2.5. Furthermore, FGFs could activate different pathways like Ras/MAPK, PI3K, or PLC, then the contribution of the Hh, Wnt, FGF, and BMP signaling pathways to the early patterning of the retina (Cardozo et al., 2020). This was in line with our study that FGF8 and FGF10 were associated with the PI3K/Akt signaling pathway and the regulation of cell proliferation. Therefore, PM2.5 exposure potentially affected cell proliferation and early retinal differentiation of hEROs.

Many studies showed PM2.5-induced oxidative stress, mitochondrial dysfunction, and inflammatory response, which is similar to cigarette smoking and maybe the main toxic mechanisms for cell/tissue damage (Li et al., 2018; Wang et al., 2019). Stem cells or stem cell derivatives can alleviate such insults (Li et al., 2017; Gao et al., 2021). This study also showed that the expression of immune-related genes, including TLR4, IL-7R, IL-1A, and CCL2, was upregulated. As immune cells, such as microglia were absent in our hEROs model, we speculated that PM2.5-induced inflammatory response might not be the main toxic mechanism.

There are several limitations to our study. First, the hEROs in our study were in the early stage of retinal development. The effect of PM2.5 on retinal development of late stage needs to be uncovered. Second, it is unlikely that the precise exposure of 100 μg/ml of PM2.5 used in the present study will occur in our daily lives. It is hard to tell the critical concentration of PM2.5 that can affect the retina in the human body. Third, the heterogeneous cell population of organoids could lead to difficulties with the interpretation of RNA-seq results. The usage of single-cell RNA-seq technologies aims to minimize this effect by isolation of cell types.

## Conclusion

In conclusion, this prospective study provides the first evidence that PM2.5 exposure over certain concentrations and periods associated with the altered development of hESC-derived retinal organoids. Despite the fact that further studies are warranted to identify the effect of PM2.5 on the human retina, our results suggest that the hEROs model may represent a powerful tool for the evaluation of the novel relationship between PM2.5 exposure and human early retinal development.

## Data Availability Statement

All datasets generated for this study are included in the article/Supplementary Material, further inquiries can be directed to the corresponding author/s.

## Author Contributions

YZ carried out the experiment, made contribution to the acquisition, analysis, and interpretation of data for the work. ML wrote the manuscript with support from YZ and TZ. TZ contributed to final approval of the version to be submitted and contributed to the experiment. QL contributed to final approval of the version to be published and analysis of data for the work. YL contributed to the interpretation of data for the work. LG contributed to measurements and assisted with experiment. SC contributed to the data analysis and critical revision of the article. HX contributed to the conception and design of the work and final approval of the version to be submitted. All authors contributed to the article and approved the submitted version.

## Conflict of Interest

The authors declare that the research was conducted in the absence of any commercial or financial relationships that could be construed as a potential conflict of interest.

## Footnotes

1 http://string-db.org/
