# Supplementary material for: The Impact of Particulate Matter (PM2.5) on Human Retinal Development in hESC-Derived Retinal Organoids
Source: Front Cell Dev Biol. 2021 Feb 12;9:607341. doi: 10.3389/fcell.2021.607341 (PMC7907455; doi:10.3389/fcell.2021.607341)
Supplement: Supplementary file 4 [file Data_Sheet_1.DOC]

**The impact of particulate matter (PM2.5) on** **human retinal development in hESC-derived retinal organoids**

**Supplementary Materials**


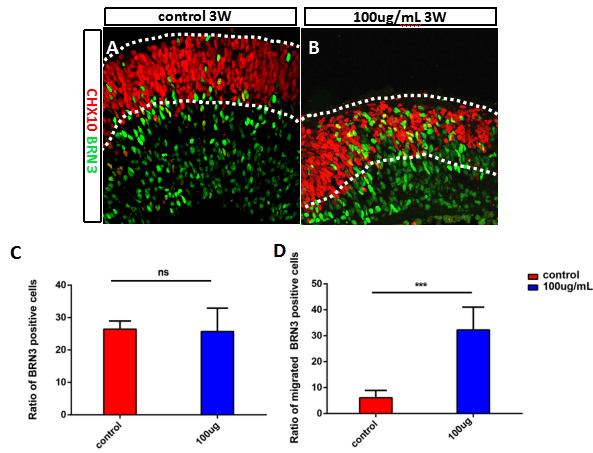


**Figure S1.** The cell migration of hERO-NRs with 100 μg/mL PM2.5 exposure. (A-B) Immunostaining analysis of RGC-related marker in the hERO-NRs (after 3 weeks, 100 μg/mL PM2.5 treatment). Co-tagging of Chx10 and BRN3 at control group and PM2.5-100 μg/mL group. Most missing Chx10-positive cells(red) express the RGC maker BRN3(green). (C) Statistical analysis of BRN3 positive cells in the whole hERO-NRs. (D) Statistical analysis of migration BRN3 positive cells in the Chx10^+^ cell region, As shown above between the white dotted lines. (NS, no significant difference, ****P* < 0.001).


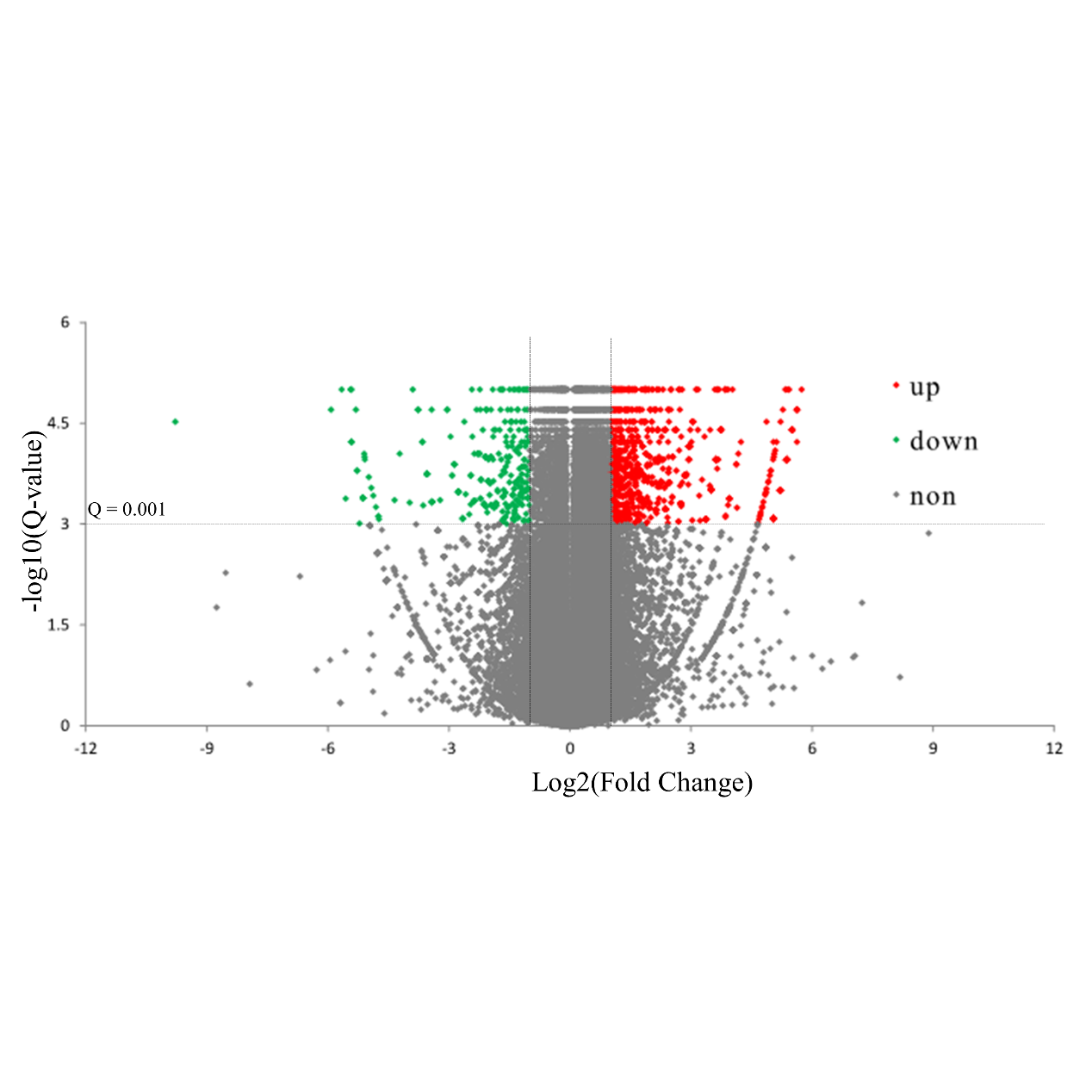


**Figure S2.** The gene ratio distribution of differentially-expressed genes between PM2.5 exposure and control groups. A red dot signifies an up-regulated gene, and a green dot signifies a down-regulated gene. Log2 Fold Change ≥ 1 and Q < 0.01 were set as cut-off values.


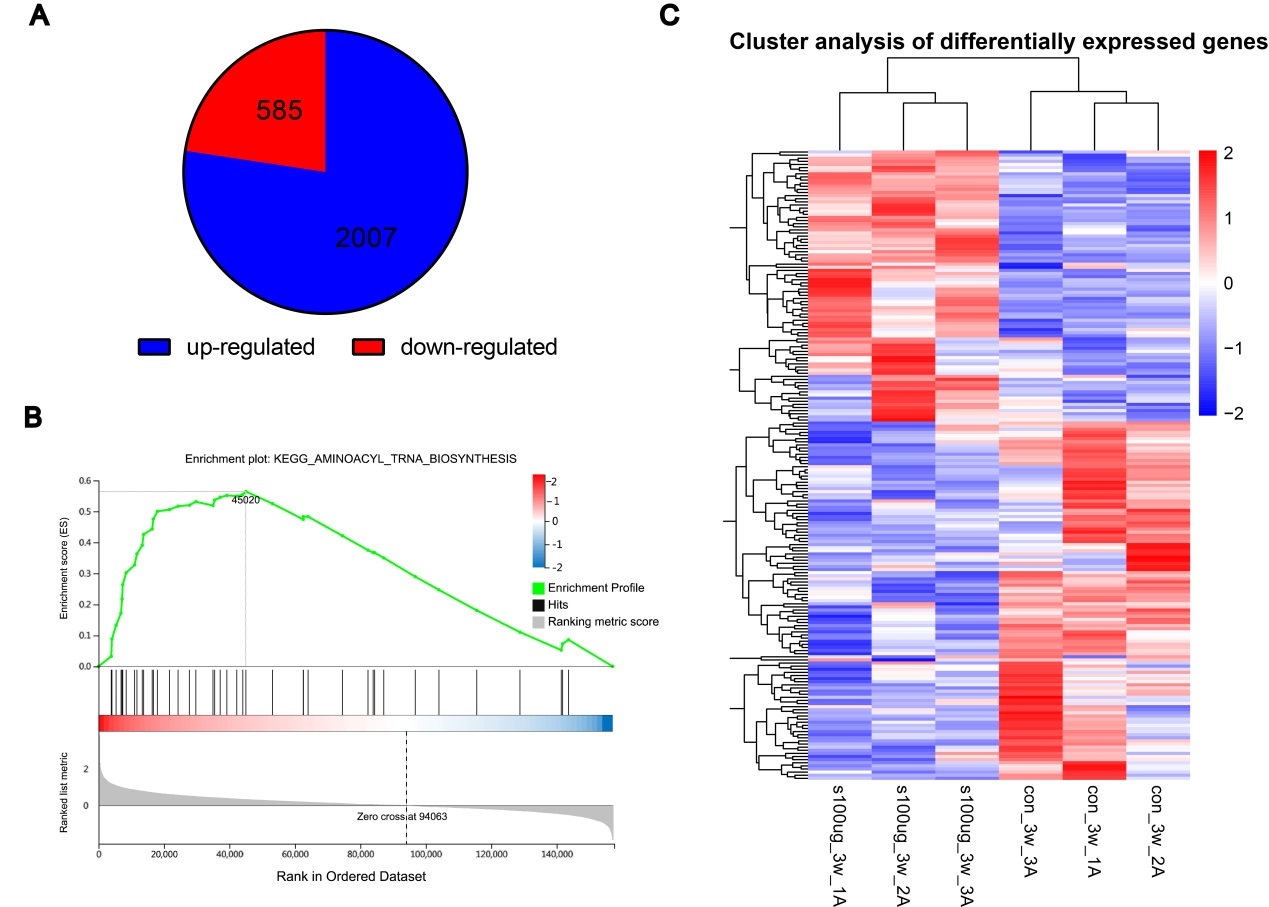


**Figure S3.** The representative significant gene clusters in GSEA analysis on all genes detected in RNA-seq.


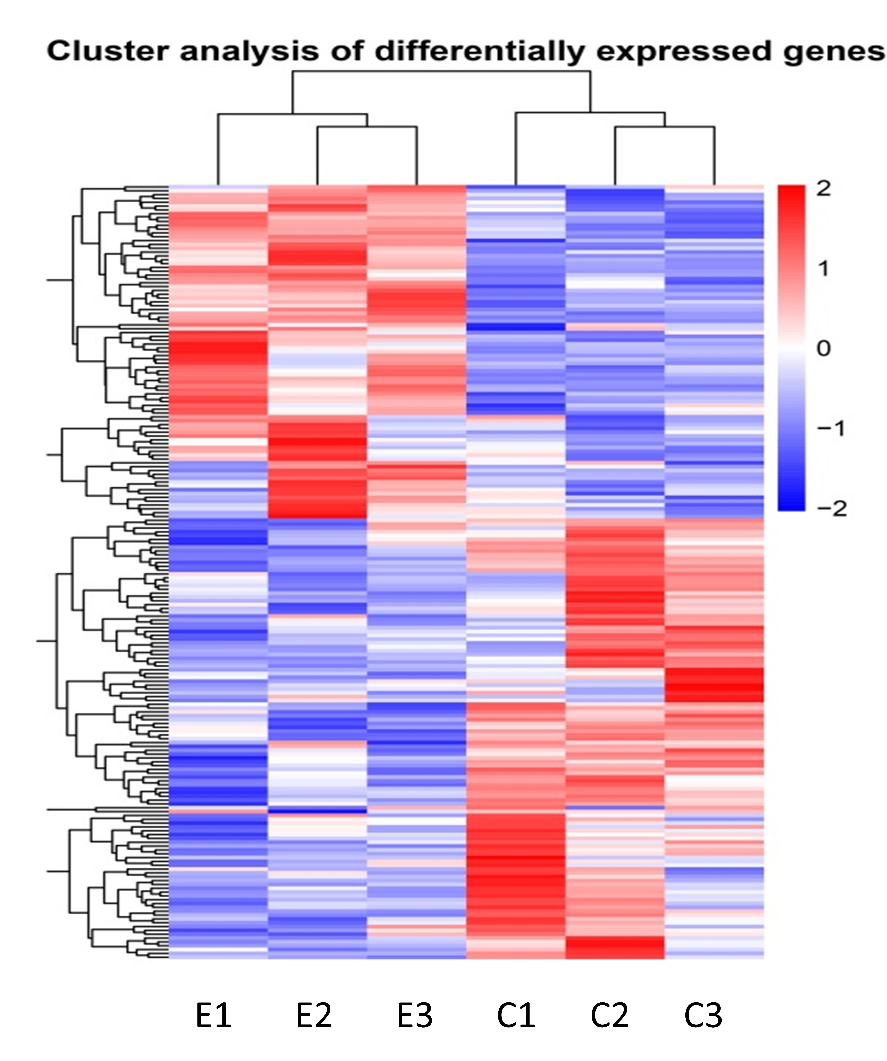


**Figure S4.** Heatmap of relative expression levels of differentially expressed genes of hEROs. Fold changes of expression have been collapsed into color scale, and red blocks indicated up-regulated DE-ExoMiRs, whereas blue blocks represented down-regulated genes. C = control groups, E = PM2.5 exposed-groups.


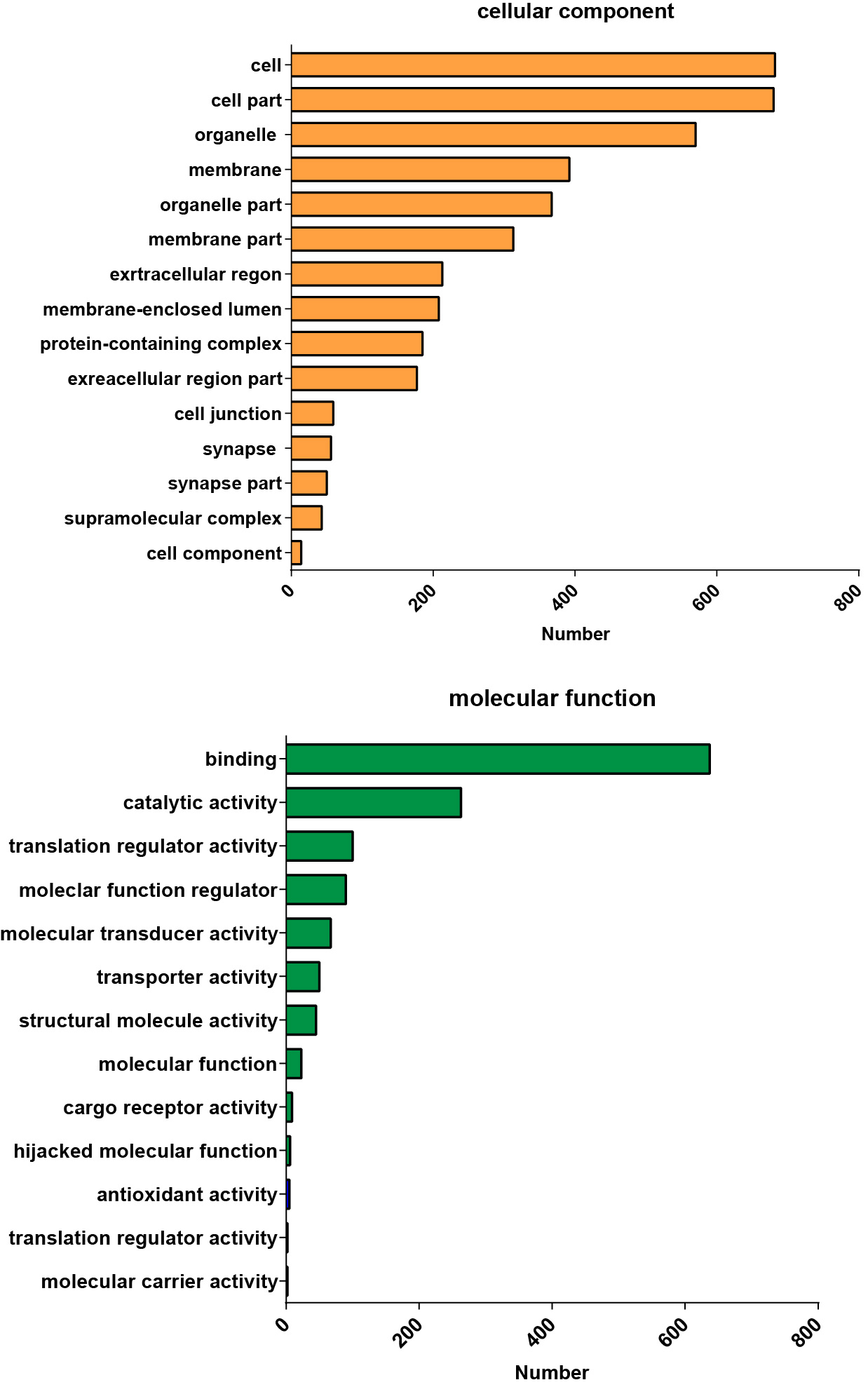


**Figure S5.** Classification of the identified DEGs into cellular components and molecular function.


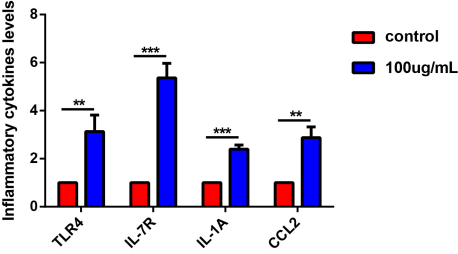


**Figure S6** Expression of imflammatory cytokines and their receptors in the PM 2.5 exposed hESC-derived retinal organoids. ***P* < 0.01, ****P* < 0.001 vs control.
